# Effects and mechanisms of 8-prenylnaringenin on osteoblast MC3T3-E1 and osteoclast-like cells RAW264.7

**DOI:** 10.1002/fsn3.109

**Published:** 2014-04-06

**Authors:** Dan Luo, Lumei Kang, Yuhui Ma, Hongping Chen, Haibin Kuang, Qiren Huang, Ming He, Weijie Peng

**Affiliations:** State Key Laboratory of Food Science and Technology, Medical School, Nanchang UniversityBayi Road 461, Nanchang, Jiangxi Province, China

**Keywords:** 8-Prenylnaringenin, daidzein, estrogen receptor, genistein, osteoblast, osteoclast

## Abstract

8-Prenylnaringenin (8-PN) is a phytoestrogen with the highest estrogenic activity. The objective of the present study was to confirm the superiority of 8-PN on bone metabolisms and the estrogen receptor (ER) subtype mediating effects of 8-PN. The osteoblast MC3T3-E1 and osteoclast-like cell line RAW264.7 were treated with 17*β*-estradiol (10^−8^ mol/L), genistein (10^−5^ mol/L), daidzein (10^−5^ mol/L), 8-PN (10^−5^ mol/L) alone or in the presence of ER*α* antagonist MPP (10^−7^ mol/L) and ER*β* antagonist PTHPP (1.5 × 10^−7^ mol/L). It has been found that 8-PN did not affect osteoblast proliferation, and that 8-PN increased alkaline phosphatase (ALP) activity, osteocalcin (OCN) concentrations, and the mineralized nodules. 8-PN inhibited RAW264.7 differentiating into osteoclasts and reduced the pit area of bone resorption. 8-PN could also inhibit the protein and mRNA expression of receptor activator of nuclear factor-*κ*B ligand (RANKL) in osteoblasts, and conversely promote the expression of osteoprotegerin (OPG). These effects of 8-PN were mainly inhibited not by PTHPP but by MPP and they were weaker than estrogen's effects but stronger than those of genistein and daidzein. In conclusion, the effects of 8-PN on promoting osteoblastic bone formation and inhibiting osteoclastic bone resorption were mediated by ER*α* instead of ER*β* and the efficacy was more potent than that of the two classic phytoestrogens: genistein and daidzein.

## Introduction

Osteoporosis, usually arising from estrogen deficiency in postmenopausal women, is a common illness in elderly women. Estrogen deficiency results in an imbalance in bone remodeling, with a substantial relative increase in osteoclastic bone resorption compared to osteoblastic bone formation (Li et al. 2009; Hu et al. 2011; Khosla et al. 2012). Estrogen replacement therapy has been well established in the treatment of postmenopausal osteoporosis for decades until recent past users of hormonal replacement therapy (HRT) were found having a higher risk of cardiovascular diseases, breast cancer, etc. (Santen et al. 2010). More and more studies emphasized on exploring alternative medicines and agents for estrogen replacement therapy such as phytoestrogens (Lagari and Levis 2012).

Phytoestrogens are a group of biological activity substances extracted from plants and seeds and they have chemical structures similar to physiological estradiol. This structural similarity accounts for these compounds binding to estrogen receptor (ER) and exerting various estrogenic and antiestrogenic effects. There are three main families of phytoestrogens: isoflavones, lignans, and coumestans. As estrogen-mimic compounds, phytoestrogens have been considered as a therapeutic option for osteoporosis. In this regard, the effects of phytoestrogens on bone metabolism have been examined in a number of cellular, animal, and clinical trials, but with inconsistent results. Most of the related reviews seem to come to the conclusion that current evidences are not sufficient to make recommendations regarding the effects of phytoestrogens on bone health. Variable results may be due to the differences in study design, duration, populations studied, and also the insufficient potential of estrogenic activities of classic isoflavoid such as genestein and daidzein (Patisaul and Jefferson 2010).

Recently, 8-Prenylnaringenin (8-PN), 8-isoprene-4,5-7-hydroxy flavanone, a demethylating fulvic phenol isomer, has aroused much interest as it is considered to be by far the most potent phytoestrogen with higher estrogenic bioactivities than others (Schaefer et al. 2003; Heyerick et al. 2006). 8-PN is an active constituent isolated from the female flowers of *Humulus lupulus* L. (hop) (Cannabaceae) which is well known for the usage of bitterness, preservative, and flavoring in beer brewing. There have been several studies performed that demonstrated the potential of 8-PN to alleviate climacteric symptoms like osteoporosis, vasomotoric complaints, and sexual motivation (Zanoli and Zavatti 2008; Keiler et al. 2013). The promising potential of 8-PN on postmenopause osteoporosis has been shown in a few cellular and animal experiments (Effenberger et al. 2005; Sehmisch et al. 2008; Pedrera-Zamorano et al. 2009; Ming et al. 2012, 2013). However, only limited data have been found about whether the effects of 8-PN on bone turnover are higher than those of other phytoestrogens (Sehmisch et al. 2008). On the other hand, although several studies applying rapid yeast estrogen bioassays stably expressing human ER have showed that 8-PN exhibited higher preference for ER*α* than for ER*β* (Milligan et al. 2002; Bovee et al. 2004; Chadwick et al. 2006), we cannot find any study about ER subtype mediating 8-PN function on bone metabolism. This study aims to compare the effects of 8-PN with genistein and daidzein on the differentiation and functions of osteoblast and osteoclast, and ascertain how the ER subtype mediates the bone effects of 8-PN by applying ER*α* or -*β* special antagonist.

## Materials and Methods

### Reagents and cell culture

Mouse osteoblastic cell line MC3T3-E1 was obtained from Peking Union Medical College Center (Beijing, China), and mouse mononuclear macrophage RAW264.7 was purchased from the Chinese Academy of Sciences (Beijing, China). Culture medium (Dulbecco's Modified Eagle's Medium [DMEM], *α*-MEM) was obtained from Invitrogen (Auckland, Scotland, UK). Fetal bovine serum (FBS) was from Lanzhou National Hycolone Bio-Engineering (Lanzhou, China). 8-PN, 17*β*-estradiol, daidaizn, and geneistein were purchased from Sigma (St. Louis, MO), and ER*α* antagonist methyl-piperidino-pyrazole (MPP) and ER*β* antagonist 4-[2-phenyl-5,7-bis (tri-fluoro-methyl) pyrazolo [1,5-a] pyrimidin-3-yl]phenol (PTHPP) were from Tocris biosciences (Bristol, UK). Receptor activator of nuclear factor-*κ*B ligand (RANKL) and macrophage colony-stimulating factor (M-CSF) were purchased from PeproTech (Rocky Hill, NJ). Osteocalcin (OCN), RANKL, and osteoprotegerin (OPG) ELISA Kit were purchased from USCN (Houston, TX). The primers were synthesized in Shanghai Bio-Engineering Technology Service Co., Ltd. (Shanghai, China). All other reagents used were of analytical grade. MC3T3-E1 cells were cultured in phenol red-free *α*-MEM medium supplemented with 10% FBS, 100 U/mL penicillin and 100 *μ*g/mL streptomycin, 50 mg/L ascorbic acid, and 2 mmol/L sodium *β*-glycerophosphate. RAW264.7 cells were maintained in DMEM supplemented with 10% FBS, 100 U/mL penicillin and 100 *μ*g/mL streptomycin, 25 ng/mL M-CSF, and 50 ng/mL RANKL. Both the cell lines were incubated at 37°C with 5% CO_2_ and saturated humidity. The medium was changed every 3 days. 0.25% trypsin digested and passaged. The cells were treated with 8-PN (10^−5^ mol/L) alone and in the presence of MPP (10^−7^ mol/L) or PTHPP (1.5 × 10^−7^ mol/L), genistein (10^−5^ mol/L, Gen), daidzein (10^−5^ mol/L, Dai), and 17*β*-estradiol (10^−8^ mol/L, E_2_) for different periods.

### MTT of MC3T3-E1 in proliferation phase

MC3T3-E1 cells were plated into 96-well plates at 10^4^ cells/well in 100 *μ*L culture medium in which FBS was replaced by 10% activated carbon-treated FBS, and cells were treated with drugs the next day. After 48 h, the cell viability was detected with MTT according to the manufacturers' instructions. In brief, each well was added with 10 *μ*L MTT [3-(4,5-dimethylthiazol-2-yl)-2,5-diphenyl tetrazolium bromide] solution (5 mg/mL) and incubated for 4 h. Discarded the culture medium, added 100 *μ*L DMSO and shook for 10 min. Then the absorption values were measured at the wavelength of 490 nm. The culture medium with no cells was used as blank control. Cell viability = (absorption values of group treated/absorption values of control group) × 100%.

### Alkaline phosphatase (ALP) activity and OCN, RANKL, and OPG ELISA analysis of MC3T3-E1 in differentiation phase

MC3T3-E1 cells were plated into 12-well plates. It almost took 6 days for MC3T3-E1 to differentiate into mature osteoblasts. After 6 days, FBS was replaced by 10% activated carbon-treated FBS for estrogen starvation for 3 days. Subsequently, agents were added into medium 3 days. The cells were treated with drugs on the 9th day. After treating for 48 or 72 h, the culture medium was collected and used for the ELISA analysis of OCN, RANKL, and OPG according to the manufacturer's instructions. Additionally, each well was added with 300 *μ*L of cell lysis buffer and placed on the ice for 30 min. After centrifuged for 10 min at 5000 rpm, the lysate supernatant was collected and ALP activity and total protein content were measured by automatic biochemical analyzer. The total protein content was measured by BCA.

### Detection of RANKL and OPG mRNA in MC3T3-E1 in differentiation phase

MC3T3-E1 cells were plated into 6-well plates and followed the same procedure as in section Alkaline phosphatase (ALP) activity and OCN, RANKL, and OPG ELISA analysis of MC3T3-E1 in differentiation phase. Agents were added into medium 3 days before RNA isolation. Total RNA was isolated with Trizol (Invitrogen) according to the manufacturer's instructions. RNA (1 *μ*g, measured photometrically) was transcribed into cDNA by Superscript Kit (Invitrogen). The sequences of specific primers, annealing temperature, PCR cycles used for investigated genes were shown in Table 1. In brief, cDNA synthesis was carried out for 60 min at 37°C in a final volume of 20 *μ*L (containing 0.5 mmol/L of each dNTP, 1 *μ*mol/L of oligo-dT starter, 0.2 U/*μ*L of Omniscript reverse transcriptase, and 0.5 U/*μ*L of RNase inhibitor). The amplification reactions were performed in a total volume of 25 *μ*L containing, apart from cDNA, 2 mmol/L of MgCl_2_, 0.25 mmol/L of each dNTP, 0.25 *μ*mol/L of each sense and antisense primer, and 0.1 U/*μ*L of Taq DNA Polymerase (Fermentas (MBI), Vilnius, Lithuania). All reactions were performed in a thermal cycler (PE). The amplified fragments were separated in 2% agarose gel by electrophoresis, stained with ethidium bromide and photographed.

**Table 1:** PCR condition for investigated genes

	Forward primer(5′–3′)	Reverse primer(5′–3′)	bp	AT (°C)	Cycles
*β*-actin	CTCCATCCTGGCCTCGCTGT	GCTGTCACCTTCACCGTTCC	436	56	25
RANKL	CCATCGGGTTCCCATAAAGT	CCCCAAAGTACGTCGCATCT	403	51	35
OPG	TGTTCCTACCAAGATTATACCAAAT	CGCTCGATTTGCAGGTCTTT	338	58	30

AT, annealing temperature; OPG, osteoprotegerin; RANKl, receptor activator of nuclear factor-*κ*B ligand.

### Detection of calcified nodules of MC3T3-E1 in mineralization phase

MC3T3-E1 cells were plated into 24-well plates in 500 *μ*L culture medium with 10% activated carbon treated FBS, and then the agents were added into the wells. The culture medium was changed every 3 days. On day 28, the calcified nodules were stained with alizarin red. The plates were washed with phosphate-buffered saline (PBS) for twice and the cells were fixed for 10 min in 95% ethanol. After the cells were washed three times in deionized water, 0.1% alizarin red-tris-HCl (pH 8.3) was added into the well and the plate was incubated for 30 min at 37°C. Then the cells were washed three times with deionized water. After air-dried and mounted, the calcified nodules were observed under the microscope.

### Osteoclast identification and count

The RAW264.7 cells were placed into 24-well plates (cells growing on glass coverslips) at 5 × 10^4^ cells/well in 500 *μ*L culture medium with 10% activated carbon-treated FBS and agents were added into the wells. The medium was changed every 3 days. After treated for 6, 9, and 12 days, the coverslips with RAW264.7 cells were taken out and rinsed once with PBS. The coverslips were fixed for 10 min in glutaraldehyde at 4°C, and washed three times with deionized water. Then the coverslips were put into the TRAP staining solution which had been preheated to 37°C, avoiding light and incubating for 1 h. After rinsed with deionized water thrice for 1 min, the coverslips were natural dried and were mounted with neutral resin. The coverslips were observed under the microscope. Cells with multinuclear (≥3) TRAP^+^ were considered to be osteoclasts. Three coverslips in each group were counted.

### Detection of the bone resorption activity of osteoclastic

Fresh bovine femoral compact bone was cross cut with low speed saws slicing machine, and the slices were 80 *μ*m thick and 20 mm × 20 mm size. The slices were then ground to 50 *μ*m thick with frosted glass and washed thrice for 10 min with an ultrasonic cleaning device. The slices were put into 75% alcohol for 24 h and washed five times with culture medium. Then, they were air-dried and exposed to UV light overnight. The RAW264.7 cells were placed into 24-well plates which contained bovine femoral slices at 5 × 10^4^ cells/well. Each well contained 500 *μ*L medium. The culture medium was DMEM supplemented with 10% normal FBS. At the differentiation phase of day 6, the normal FBS was changed into FBS treated with 10% activated carbon. At the differentiation phase of day 9 (estrogen starvation for 3 days), the agents were added into the wells. On day 15, the stain was performed. The bone slices attached with cells were taken out and fixed in the 2.5% glutaraldehyde for 10 min at 4°C. Ultrasonic cleaning was done thrice for 3 min with 0.25 mol/L ammonia hydroxide and washed twice with deionized water. They were air-dried and mounted with neutral resin. The bone pits (in classic shape, such as round, oval, and sausage and shown as blue metachromatic area with clear border) were observed under optical microscope. The area of the bone pits was calculated by the IPP software (version 5; Media Cybernetics, Inc., Rockville, MD). Three slices were used for analysis at each time point.

### Data analysis

Data were presented as the mean ± SD. Analysis of variance (ANOVA) for overall comparison was followed by LSD for paired comparisons. All statistical analyses were carried out with the SPSS 11.5 software (SPSS Inc., Chicago, IL). A value of *P* < 0.05 was considered statistically significant.

## Results

### Effects on the proliferation of MC3T3-E1 cell in proliferation phase

**Figure 1:**
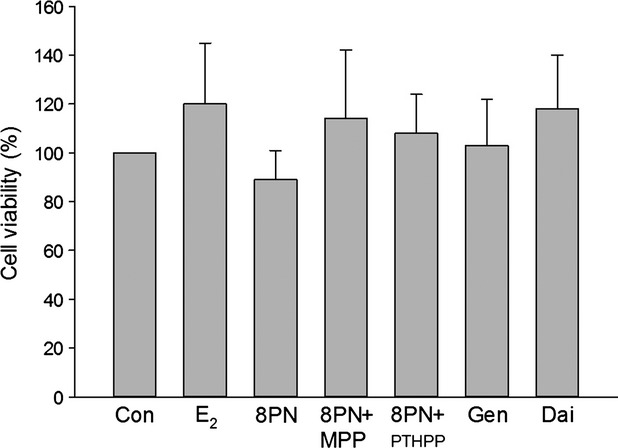
Effects of 48 h treatment of 17*β*-estradiol (E_2_, 10^−8^ mol/L), genistein (Gen, 10^−5^ mol/L), daidzein (Dai, 10^−5^ mol/L), and 8-prenylnaringenin (8PN, 10^−^5 mol/L) alone or supplemented with ER*α* antagonist methyl-piperidino-pyrazole (MPP, 10^−7^ mol/L) or ER*β* antagonist 4-[2-phenyl-5,7-bis (tri-fluoro-methyl) pyrazolo [1,5-a] pyrimidin-3-yl] phenol (PTHPP, 1.5 × 10^−7^ mol/L) on the number of MC3T3-E1 cells. Con, control group; ER, estrogen receptor.

As shown in Figure 1, on day 2 of the proliferation phase, MC3T3-E1 cells were treated with drugs for 48 h. Cell proliferation was detected by MTT. The results indicated that there were no significant differences among the effects of drugs on the cell proliferation.

### Effects on the differentiation of MC3T3-E1

**Figure 2:**
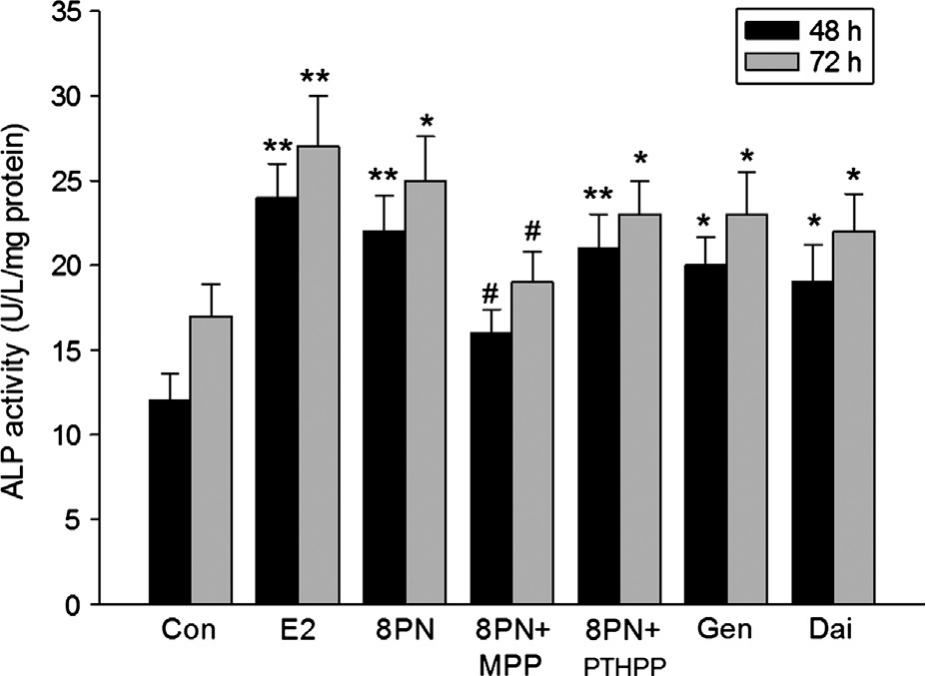
Effects of 48 or 72 h treatment of 17*β*-estradiol (E_2_, 10^−8^ mol/L), genistein (Gen, 10^−5^ mol/L), daidzein (Dai, 10^−5^ mol/L), and 8-prenylnaringenin (8PN,10^−5^ mol/L) alone or supplemented with ER*α* antagonist methyl-piperidino-pyrazole (MPP, 10^−7^ mol/L) or ER*β* antagonist 4-[2-phenyl-5,7-bis (tri-fluoro-methyl) pyrazolo [1,5-a] pyrimidin-3-yl] phenol (PTHPP, 1.5 × 10^−7^ mol/L) on the alkaline (ALP) activity (U/L) of MC3T3-E1. *P < 0.05 and **P < 0.01 versus control group (Con), #*P* < 0.05 versus 8PN group. ER, estrogen receptor; ALP, alkaline phosphatase.

**Figure 3:**
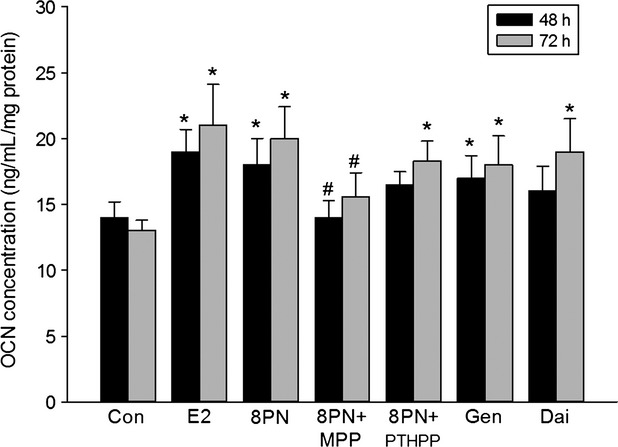
Effects of 48 or 72 h treatment of 17*β*-estradiol (E_2_, 10^−8^ mol/L), genistein (Gen, 10^−5^ mol/L), daidzein (Dai, 10^−5^ mol/L), and 8-prenylnaringenin (8PN, 10^−5^ mol/L) alone or supplemented with ER*α* antagonist methyl-piperidino-pyrazole (MPP, 10^−7^ mol/L) or ER*β* antagonist 4-[2-phenyl-5,7-bis (tri-fluoro-methyl) pyrazolo [1,5-a] pyrimidin-3-yl] phenol (PTHPP, 1.5 × 10^−7^ mol/L) on the osteocalcin (OCN) concentration (ng/mL) of MC3T3-E1. *P < 0.05 versus control group (Con). #*P* < 0.05 versus 8PN group. ER, estrogen receptor.

The MC3T3-E1 cells were treated with 8-PN and other agents on day 9 of differentiation phase. As shown in Figure 2, 8-PN at 10^−5^ mol/L increased ALP activity of MC3T3-E1 equivalent to E_2_ at 10^−8^ mol/L after 48 or 72 h of administration. MPP inhibited the effects of 8-PN (*P* < 0.05), whereas PTHPP had no influences on the effects of 8-PN. It was shown that the tendency was enhanced in the 8-PN group compared with in the genistein and daidzein groups at the same concentration (*P* > 0.05). The changes of OCN level among groups were similar to those of ALP levels (Fig. 3).

### Effects on the calcified nodule of MC3T3-E1 in mineralization

**Figure 4:**
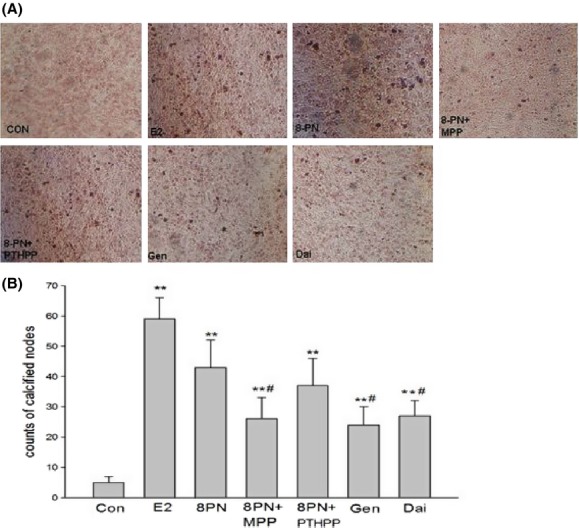
(A) Effects of 17*β*-estradiol (E_2_, 10^−8^ mol/L), genistein (Gen, 10^−5^ mol/L), daidzein (Dai, 10^−5^ mol/L), and 8-prenylnaringenin (8PN, 10^−5^ mol/L) alone or supplemented with ER*α* antagonist methyl-piperidino-pyrazole (MPP, 10^−7^ mol/L) or ER*β* antagonist 4-[2-phenyl-5,7-bis (tri-fluoro-methyl) pyrazolo [1,5-a] pyrimidin-3-yl] phenol (PTHPP, 1.5 × 10^−7^ mol/L) on mineralized nodules of MC3T3-E1 demonstrated by alizarin red staining at day 28 (100×). (B) Different counts of calcified nodes among groups. **P < 0.01 versus control group (Con), #*P* < 0.05 versus 8PN group. ER, estrogen receptor.

As shown in Figure 4, MC3T3-E1 cells were treated long-term with 8-PN and other agents. On day 28 of mineralization, alizarin red staining was performed for detecting calcified nodule. E_2_ and phytoestrogens promoted the formation of the calcified nodules. The effects of 8-PN seemed to be inhibited not by PTHPP but by MPP. Additionally, there seemed to be more calcified nodules in 8-PN groups than in genistein and daidzein groups.

### Effects on osteoclast differentiation

**Figure 5:**
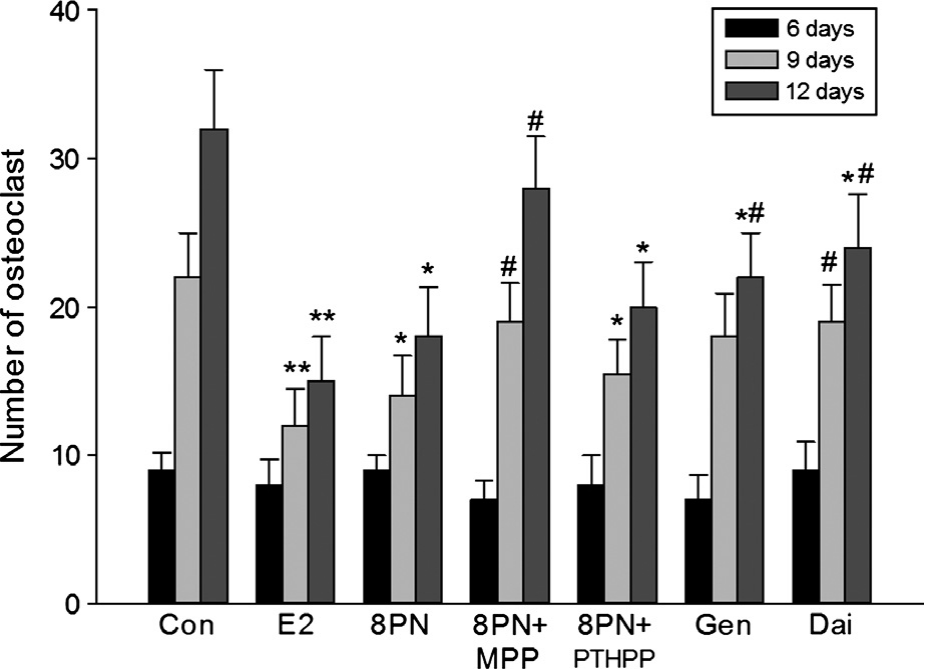
Effects of 17*β*-estradiol (E_2_, 10^−8^ mol/L), genistein (Gen, 10^−5^ mol/L), daidzein (Dai, 10^−5^ mol/L), and 8-prenylnaringenin (8PN, 10^−5^ mol/L) alone or supplemented with ER*α* antagonist methyl-piperidino-pyrazole (MPP, 10^−7^ mol/L) or ER*β* antagonist 4-[2-phenyl-5,7-bis (tri-fluoro-methyl) pyrazolo [1,5-a] pyrimidin-3-yl] phenol (PTHPP, 1.5 × 10^−7^ mol/L) on the number of osteoclast-like cells characterized via tartrate-resistant acid phosphatase (TRAP) staining from RAW264.7 induced by macrophage colony-stimulating factor (M-CSF) and receptor activator for nuclear factor-*κ* B ligand (RANKL) for 6, 9, 12 days (×200). *P < 0.01 and **P < 0.01 vs. control group (Con), #*P* < 0.05 versus 8PN group. ER, estrogen receptor.

On the third, sixth, ninth, and twelfth day after the treatment, the RAW264.7 cells were observed under the microscope. On day 3, there were no osteoclasts appeared. As shown in Figure 5, on day 6, there were a few osteoclasts but no significant differences among the groups. On day 9, there were less osteoclasts in the E_2_ group (*P* < 0.01), 8-PN group (*P* < 0.05) than those in the control group. And on day 12, the number of osteoclasts was obviously decreased in the E_2_ (*P* < 0.01), 8-PN (*P* < 0.05), genistein (*P* < 0.05), and daidzein groups (*P* < 0.05) when compared with those in the control group. Likewise, the effects of 8-PN were equivalent to those of E_2_ and significantly stronger than those of Gen and Dai (*P* < 0.05) and were inhibited not by PTHPP but by MPP (*P* < 0.05).

### Effects on osteoclast activity

**Figure 6:**
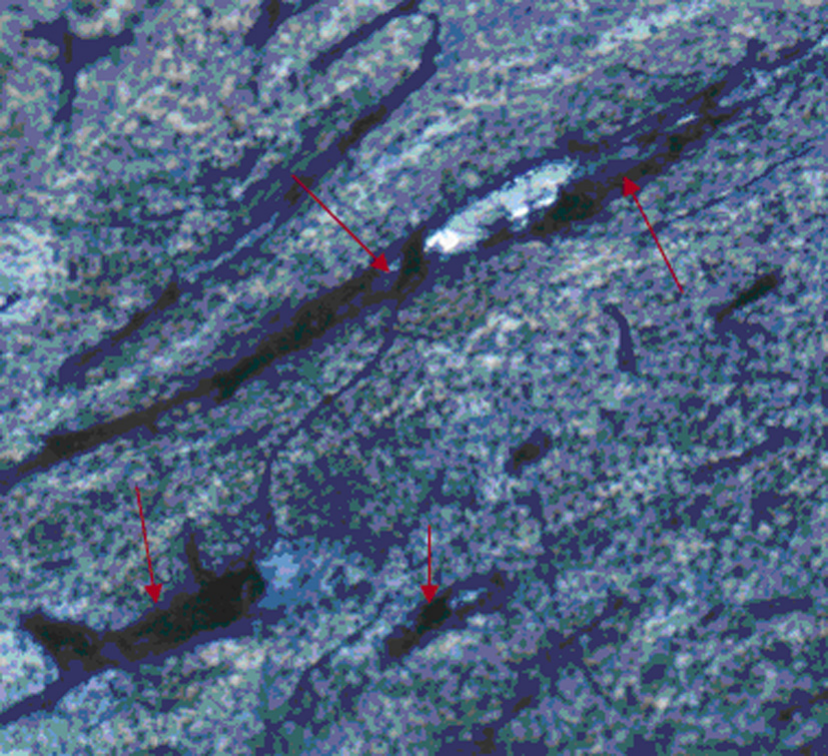
The characteristics of bone resorption pits on bone slices as shown by arrows (400×).

**Figure 7:**
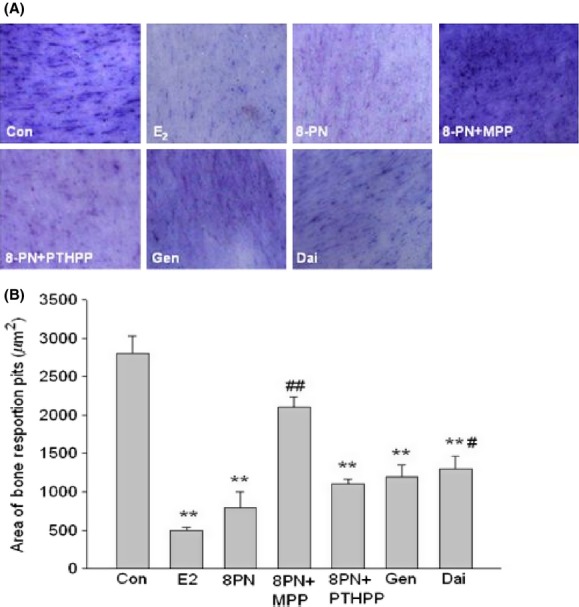
(A) Effects of 17*β*-estradiol (E_2_, 10^−8^ mol/L), genistein (Gen, 10^−5^ mol/L), daidzein (Dai, 10^−5^ mol/L), and 8-prenylnaringenin (8PN, 10^−5^ mol/L) alone or supplemented with ER*α* antagonist methyl-piperidino-pyrazole (MPP, 10^−7^ mol/L) or ER*β* antagonist 4-[2-phenyl-5,7-bis (tri-fluoro- methyl) pyrazolo [1,5-a] pyrimidin-3-yl] phenol (PTHPP, 1.5 × 10^−7^ mol/L) on bone resorption pits on bone slice observed under optical microscope (100×). (B) Different area of bone resorption pits calculated by the IPP software among groups. **P < 0.01 versus control group (Con), #*P* < 0.05 and ##*P* < 0.01 versus 8PN group. ER, estrogen receptor.

RAW264.7 cells induced osteoclastic differentiation with the stimulation of RANKL and m-CSF. On day 15, after the bone slices were added, the blue bone absorption pits appeared in the round, oval, and sausage shaped (Fig. 6). The borders of the bone pits were clear and the sizes were different. When treated with the compounds, the bone slices coculturing with RAW264.7 were shown differently decreased area of the bone pits. The effects of 8-PN were similar to those of E_2_ and significantly stronger than those of Gen and Dai (*P* < 0.05) and were inhibited not by PTHPP but by MPP(*P* < 0.05) (Fig. 7).

### Effects on the expressions of RANKL and OPG in MC3T3-E1 cells

**Figure 8:**
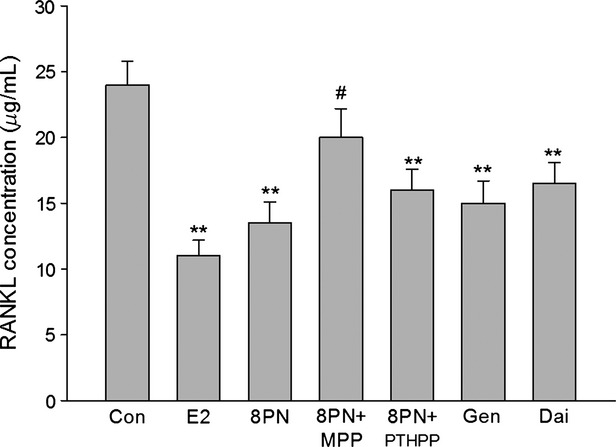
Effects of 17*β*-estradiol (E_2_, 10^−8^ mol/L), genistein (Gen, 10^−5^ mol/L), daidzein (Dai, 10^−5^ mol/L), and 8-prenylnaringenin (8PN, 10^−5^ mol/L) alone or supplemented with ER*α* antagonist methyl-piperidino-pyrazole (MPP, 10^−7^ mol/L) or ER*β* antagonist 4-[2-phenyl-5,7-bis (tri-fluoro-methyl) pyrazolo [1,5-a] pyrimidin-3-yl] phenol (PTHPP, 1.5 × 10^−7^ mol/L) on the secretion of receptor activator for nuclear factor-*κ* B ligand (RANKL) protein in MC3T3-E1 cells. **P < 0.01 versus control group (Con), #*P* < 0.05 versus 8PN group. ER, estrogen receptor.

**Figure 9:**
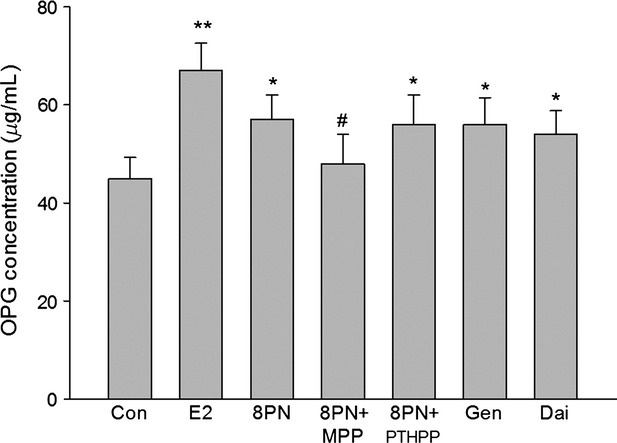
Effects of 17*β*-estradiol (E_2_, 10^−8^ mol/L), genistein (Gen, 10^−5^ mol/L), daidzein (Dai, 10^−5^ mol/L), and 8-prenylnaringenin (8PN, 10^−5^ mol/L) alone or supplemented with ER*α* antagonist methyl-piperidino-pyrazole (MPP, 10^−7^ mol/L) or ER*β* antagonist 4-[2-phenyl-5,7-bis (tri-fluoro-methyl) pyrazolo [1,5-a] pyrimidin-3-yl] phenol (PTHPP, 1.5 × 10^−7^ mol/L) on the secretion of osteoprotegerin (OPG) protein in MC3T3-E1 cells. *P < 0.05 and **P < 0.01 versus control group (Con), #*P* < 0.05 versus 8PN group. ER, estrogen receptor.

**Figure 10:**
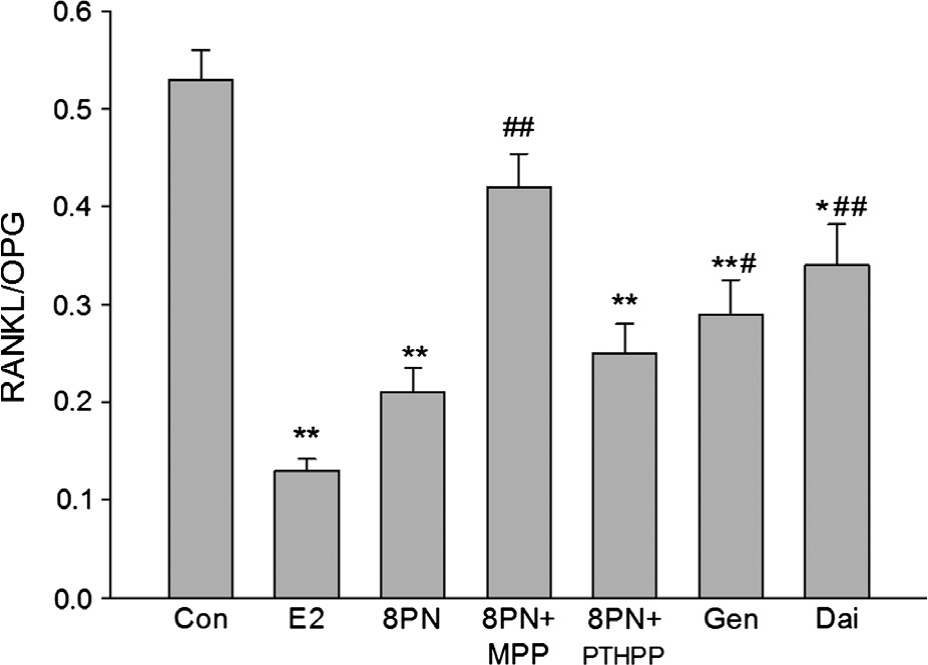
Effects of 17*β*-estradiol (E_2_, 10^−8^ mol/L), genistein (Gen, 10^−5^ mol/L), daidzein (Dai, 10^−5^ mol/L), and 8-prenylnaringenin (8PN, 10^−5^ mol/L) alone or supplemented with ER*α* antagonist methyl-piperidino-pyrazole (MPP, 10^−7^ mol/L) or ER*β* antagonist 4-[2-phenyl-5,7-bis (tri-fluoro-methyl) pyrazolo [1,5-a] pyrimidin-3-yl] phenol (PTHPP, 1.5 × 10^−7^ mol/L) on the relative value of secreting protein of receptor activator for nuclear factor-*κ*B ligand (RANKL) to osteoprotegerin (OPG) in MC3T3-E1 cells. *P < 0.05 and **P < 0.01 versus control group (Con), #*P* < 0.05 and ##*P* < 0.01 versus 8PN group. ER, estrogen receptor.

**Figure 11:**
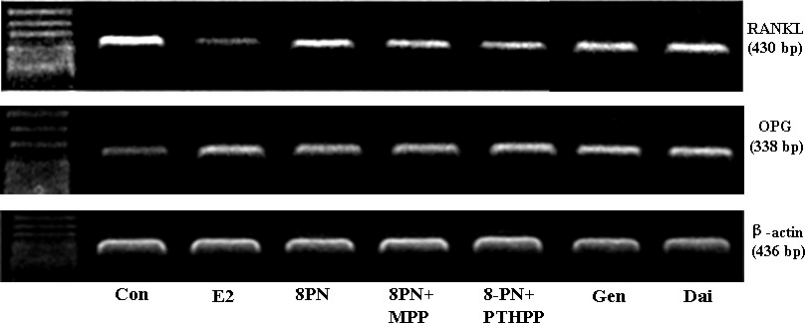
Effects of 17*β*-estradiol (E_2_, 10^−8^ mol/L), genistein (Gen, 10^−5^ mol/L), daidzein (Dai, 10^−5^ mol/L), and 8-prenylnaringenin (8PN, 10^−5^ mol/L) alone or supplemented with ER*α* antagonist methyl-piperidino-pyrazole (MPP, 10^−7^ mol/L) or ER*β* antagonist 4-[2-phenyl-5,7-bis (tri-fluoro-methyl) pyrazolo [1,5-a] pyrimidin-3-yl] phenol (PTHPP, 1.5 × 10^−7^ mol/L) on the expression of receptor activator for nuclear factor-*κ*B ligand (RANKL) and osteoprotegerin (OPG) mRNA in MC3T3-E1 cells. ER, estrogen receptor.

As shown in Figures 8–10, all of the compounds decreased the expression of RANKL protein in MC3T3-E1 cells. The effects of 8-PN were stronger than those of genistein and daidzein at the same concentration (*P* < 0.05) and were almost abolished by MPP but not by PTHPP (*P* < 0.05). All agents increased the secretion of OPG protein when compared with the control(*P* < 0.05)and there were no significant differences among the treated groups. The results of the RANKL/OPG ratio were similar to the secretion of RANKL protein. The effect of drugs on the expressions of RANKL and OPG mRNA were similar to the protein secretion (Fig. 11).

## Discussion

Effects of 8-PN on bone metabolism have been reported. Epidemiological surveys have shown that the quantitative ultrasound values of phalangeal bone were higher in the women with long-term beer consumption than the women with non-beer consumption (Pedrera-Zamorano et al. 2009). Ovariectomized rats treated with 8-PN for 12 weeks showed obviously improved bone biomechanical characteristics and bone mineral density (Sehmisch et al. 2008). 8-PN also enhanced ALP activity of human osteoblasts hFOB/ER*α*9 and increased ALP gene transcription level of human osteosarcoma cells U-2 OS/ER *α* and U-2 OS/ER*β* (Effenberger et al. 2005). In addition, 8-PN promoted bone marrow stromal cells differentiation into osteoblasts and mineralization, boosted osteoclasts apoptosis and inhibited osteoclasts differentiation (Ming et al. 2012, 2013).

In the present study, 8-PN was shown enhancing ALP activity, increasing OCN protein levels, and promoting calcification nodule formation of osteoblast cell line MC3T3-E1. On the other hand, 8-PN reduced the osteoclastic differentiation from multinuclear macrophage RAW264.7 and inhibited bone resorption shown as decreased lacunae area in bone slices. Additionally, osteoclast differentiation and maturation could not be separated from the bone microenvironment in where osteoblasts, osteocyte, bone marrow stromal cells, fibroblasts, and other cells could regulate osteoclast differentiation and function by secreting cytokines such as RANKL, OPG, and M-CSF, and so on. Higher RANKL/OPG ratio contributed to promote the differentiation of osteoclasts and bone resorption, and vice versa (Yasuda 2006). In this study, RANKL/OPG ratio in MC3T3-E1 culturing medium was less in 8-PN group than in control group since 8-PN induced secretion of OPG and inhibited RANKL secretion, which suggested that 8-PN may regulate osteoclasts indirectly via osteoblasts MC3T3-E1 besides directly regulate the differentiation and function of osteoclasts. The effects of 8-PN on osteoblast MC3T3-E1 and osteoclast-like RAW264.7 mentioned above were abolished mainly by ER*α*-selective antagonist MPP but not ER*β*-selective antagonist PTHPP, and the effects of 8-PN were weaker than those of estrogen, but stronger than those of genistein and daidzein.

It has been confirmed that 8-PN performed estrogen-like role via binding the ER in breast, endometrium and bone (Roelens et al. 2006). For the two subtypes of ER, ER*α* and ER*β*, rapid yeast estrogen bioassays stably expressing human ERs have showed that 8-PN exhibited higher preference for ER*α* than for ER*β* (Bovee et al. 2004). Our results confirmed that ER*α*, not ER*β*, mainly mediated the effects of 8-PN on promoting osteoblastic bone formation and inhibiting osteoclastic bone resorption. Additionally, it has been reported that estrogen may inhibit bone resorption and promote bone formation mainly mediated by ER*α* and the role of ER*β* remains unclear or just negatively modulating the effects of ER*α* in those processes (Barkhem et al. 1998; Windahl et al. 2001; Almeida et al. 2013). Meanwhile, the efficacy of daidzein, genistein, or other phytoestrogens has also been reported to be similar on the two subtypes or even stronger on ER*β* than on ER*α* (Barkhem et al. 1998; Bovee et al. 2004). The estrogen-like activity of 8-PN was about an order of magnitude stronger than other phytoestrogens and 1–2 orders of magnitude weaker compared to estrogen (Barkhem et al. 1998; Milligan et al. 2002; Bovee et al. 2004). Our results were similar to these studies. So the special ER selectivity and efficacy might also explain that effects of 8-PN on bone were weaker than those of estrogen, but stronger than those of other phytoestrogens. It was found that the stimulation of 8-PN on uterine and endometrial had been 10 times lower than E2 under the corresponding dose of bone protection (Humpel et al. 2005). Additionally, 8-PN could inhibit breast cancer cells MCF-7 proliferation, regulate blood lipid, be anti-inflammatory, and protect cardiovascular (Heyerick et al. 2006; Böttner et al. 2008; Brunelli et al. 2009; Paoletti et al. 2009). Our findings as well as results of other research suggested that 8-PN could be a more valuable candidate for the prevention and the treatment of postmenopausal osteoporosis than other phytoestrogens, although it needed to be further confirmed.

In conclusion, the present study suggested that phytoestrogen 8-PN promotes osteoblastic MC3T3-E1 differentiation and maturation, directly inhibits mononuclear macrophage RAW264.7 osteoclast differentiation and bone resorption, and also indirectly controls osteoclasts by regulating the expression and secretion of OPG and RANKL. These effects of 8-PN were mediated not by ER*β* but by ER*α* and were stronger than those of genistein and daidzein.
